# Joint analysis of transcriptional metabolism for flavonoid synthesis during different developmental periods in oil palm exocarp

**DOI:** 10.3389/fpls.2025.1530673

**Published:** 2025-03-24

**Authors:** Ruimin Zhang, Jerome Jeyakumar John Martin, Xiaoyu Liu, Xinyu Li, Lixia Zhou, Rui Li, Xiaopeng Fu, Wenrao Li, Hongxing Cao

**Affiliations:** ^1^ National Key Laboratory of Germplasm Innovation and Utilization of Fruit and Vegetable Horticultural Crops, College of Horticulture and Forestry, Huazhong Agricultural University, Wuhan, China; ^2^ Coconut Research Institute, Chinese Academy of Tropical Agricultural Sciences, Wenchang, China; ^3^ School of Life Sciences, Henan University, Kaifeng, Henan, China

**Keywords:** oil palm, metabolomics, transcriptomics, flavonoids, biosynthesis

## Abstract

To identify candidate genes for breeding oil palm varieties with high flavonoid content through molecular biotechnology, this study analyzed the metabolomes and transcriptomes of oil palm exocarp at different developmental stages using LC-MS/MS and RNA-Seq techniques. The green fruiting type (FS) oil palm exocarp at 95 days (FS1), 125 days (FS2), and 185 days (FS3) after pollination served as the materials. The enzyme genes F3H, CHS, ANS, and DFR were positively correlated with Quercetin-3-O-sambubioside. DFR also showed positive correlations with Afzelechin, Epiafzelechin, and Baimaside. In contrast, F3H, CHS, and ANS were negatively correlated with Hesperetin-7-O-glucoside. Additionally, CYP73A, UGT73C6, FG2-1, and FG2-2 were negatively correlated with Afzelechin, Epiafzelechin, Quercetin-3-O-sambubioside, and Baimaside, while CYP75A was negatively correlated with Epiafzelechin, Quercetin-3-O-sambubioside, and Baimaside. These results suggest that F3H, CHS, ANS, and DFR play a role in promoting Quercetin-3-O-sambubioside* synthesis, with DFR further enhancing the production of Afzelechin, Epiafzelechin, and Baimaside. On the other hand, F3H, CHS, and ANS may inhibit Hesperetin-7-O-glucoside synthesis. Meanwhile, CYP73A, UGT73C6, FG2-1, and FG2-2 appear to suppress the synthesis of multiple flavonoids, including Afzelechin, Epiafzelechin, Quercetin-3-O-sambubioside*, and Baimaside. Lastly, CYP75A is implicated in suppressing Epiafzelechin, Quercetin-3-O-sambubioside*, and Baimaside synthesis. These findings provide a foundation for future molecular breeding efforts targeting flavonoid-rich oil palm varieties.

## 1 Introduction

The oil palm (Elaeis guineensis Jacq.), a member of the palm family (Palmae), is a perennial tree native to South and Central America. As one of the most important woody oilseed crops in tropical regions, it has high economic value due to its efficient oil production. Its primary products include palm oil, extracted from the mesocarp, and palm kernel oil, derived from the kernels, both of which have extensive applications in the food, chemical, and bioenergy industries (John Martin et al., 2022). While research on oil palm has extensively focused on lipid metabolism and stress responses, the control of flavonoid biosynthesis in the fruit remains relatively unexplored. Flavonoids are vital secondary metabolites responsible for pigmentation, plant defense mechanisms, and have implications for human health due to their antioxidant properties. In oil palm, anthocyanins are a significant subclass of flavonoids found in the exocarp, contributing to the fruit’s coloration and maturity indicators. The biosynthesis of flavonoids occurs via complex metabolic pathways and is regulated by various genes and transcription factors (Yang et al., 2024b).

Flavonoids have diverse biological functions, including antioxidant and antimicrobial properties. For instance, the high antioxidant capacity of blueberries is attributed to their flavonoid content (Kalt et al., 2020). Studies have shown that flavonol glycosides are related to the astringency and bitterness of tea, with polyphenol oxidase (PPO) playing a dominant role in catalyzing flavonol glycosides in tea leaves (Guo et al., 2021). Additionally, polyphenol oxidase in walnuts exhibits resistance to environmental stress (Khodadadi et al., 2020). In rice, flavonoids like naringenin confer resistance to bacterial pathogens, while sakuranetin imparts resistance to fungal pathogens (Murata et al., 2020). Additionally, flavonoids contribute to pigmentation, such as anthocyanin accumulation in grape skins, which enhances pigmentation (Jiu et al., 2022). In root growth, flavonoids play a role in promoting development through the stimulation of flavonol biosynthesis in Arabidopsis thaliana (Tan et al., 2019). Moreover, flavonoids are involved in pollen development and contribute to the promotion of self-incompatibility during pollination in Brassica oleracea (Lan et al., 2017). High levels of flavonoid oil palm may help protect against a wide range of diseases, for example, in corn, high levels of flavonoids have powerful anti-inflammatory and anti-cancer activity (Casas et al., 2014). Flavonoids found in citrus fruits have considerable nutritional value in the treatment of cardiovascular disease (Testai and Calderone, 2017).

**Figure 1:**
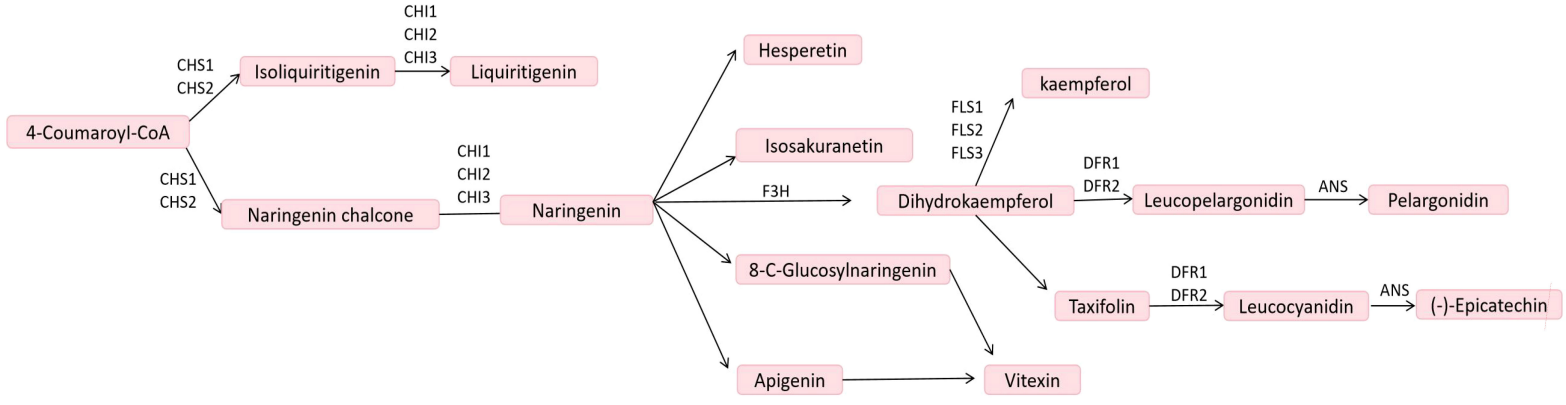
Flavonoid biosynthetic pathway (Zhang et al., 2024). CHS, chalcone synthase; CHI, chalcone isomerase; F3H, flavanone 3-hydroxylase; FLS, flavonol synthase; DFR, dihydroflavonol reductase; ANS, anthocyanidin synthase.

Flavonoids represent the largest group of polyphenolic compounds, comprising approximately 8,000 distinct flavonoid metabolites. Flavonoids can be categorized into the following main types: chalcones, flavanones, flavanonols, flavonoids,flavonols, flavanols, isoflavones, and more (Shen et al., 2022). Studies have demonstrated that oil palm possesses a high flavonoid content (Zain et al., 2021). Flavonoid biosynthesis is a complex metabolic process that primarily occurs through the phenylpropanoid pathway, converting the amino acid phenylalanine into various flavonoid compounds. This pathway is crucial for the production of numerous secondary metabolites that play significant roles in plant physiology, protection, and interactions with the environment. The figure depicts the flavonoid biosynthesis pathway, starting from 4-Coumaroyl-CoA, a precursor in the phenylpropanoid pathway. The enzyme Chalcone Synthase (CHS) catalyzes its conversion into chalcones such as Isoliquiritigenin and Naringenin chalcone, which are then cyclized by Chalcone Isomerase (CHI) into flavanones Liquiritigenin and Naringenin, respectively. Naringenin serves as a central intermediate, branching into various products: Hesperetin, Isosakuranetin, 8-C-Glucosyl-naringenin, and Apigenin, which can further glycosylate into Vitexin. Through the action of Flavanone 3-Hydroxylase (F3H), Naringenin converts into Dihydrokaempferol, which serves as a precursor for different flavonoids. Kaempferol is synthesized by Flavonol Synthase (FLS), while Leucopelargonidin, a precursor for anthocyanin Pelargonidin, forms via Dihydroflavonol Reductase (DFR) and Anthocyanidin Synthase (ANS). Taxifolin is another derivative that can lead to Leucocyanidin and eventually (-)-Epicatechin through similar enzymatic steps. These flavonoids contribute to pigmentation, UV protection, and stress responses, with the pathway tightly regulated by enzymes like CHS, CHI, F3H, FLS, DFR, and ANS (Zhang et al., 2024) (**Figure 1**). In the biosynthetic pathway of flavonoids and flavonols, all three differential metabolites—kaempferol, lignans, and quercetin—were down-regulated. Additionally, eight differential enzyme genes were identified within the pathway, among which F3’H was found to be associated with the differential metabolites (Yang, 2023). Regulation of flavonoid biosynthesis is intricately controlled by several transcription factors, including the R2R3-MYB, bHLH, and WD40 protein families. These proteins form complexes that determine the expression patterns of the biosynthesis genes, enabling the plants to adapt their flavonoid profiles according to developmental and environmental cues. For example, in *Arabidopsis*, the TT2, TT8, and TTG1 mix to regulate proanthocyanidin biosynthesis, demonstrating the complexity of regulatory mechanisms across different species (Falcone Ferreyra et al., 2012). In another study, flavonoid biosynthesis appears to involve specific transcription factors, notably AcMYB5 and AcMYB194, which have been annotated as potential regulators in the biosynthetic pathway (Lai et al., 2023).

Currently, transcriptomics and metabolomics play a very crucial role in the cultivation of various plant species (Sadat-Hosseini et al., 2020; Zhou et al., 2024). The study of different metabolites and genes involved in the flavonoid synthesis pathway in the exocarp of oil palm fruits is still in the preliminary stage. Therefore, this study hypothesizes that five differential metabolites and nine enzyme genes play a pivotal role in flavonoid synthesis in the exocarp of oil palm. By integrating transcriptomic and metabolomic data from the exocarp of green-fruited oil palm (FS) at various developmental stages, this research aims to identify key candidate genes and metabolites involved in flavonoid biosynthesis. The findings of this study will contribute valuable insights for the future cultivation and utilization of oil palm varieties, as well as for the identification and development of high-flavonoid resources and products.

## 2 Materials and methods

### 2.1 Experimental materials

The experimental materials used in this study were derived from the green-fruited oil palm variety (O×G Amazon), cultivated at the Coconut Research Institute of the Chinese Academy of Tropical Agricultural Sciences Oil Palm Research Base, located in Wenchang City, Hainan Province, China (19°33’N, 110°47’E). Fruit exocarp was harvested at three different developmental stages: 95 days after pollination (early fruit development, FS1), 125 days (rapid fruit accumulation, FS2) and 185 days (stable fruit development, FS3). At each developmental stage, three biological replicates were collected. Each replicate consisted of pooled exocarps from a minimum of 10 fruits to ensure representation of the biological variation. The exocarps were carefully peeled from the fruit and immediately flash-frozen in liquid nitrogen, then stored at -80°C for subsequent analyses.

### 2.2 Experimental procedures

#### 2.2.1 Determination and analysis of flavonoid metabolites

For metabolomic analysis, the frozen exocarp samples were ground into a fine powder using a liquid nitrogen-cooled mortar and pestle. The powdered samples were extracted with a solvent mixture of n-hexane, acetone, and ethanol in a 1:1:1 (v/v/v) ratio. The extraction was performed at room temperature for 30 minutes, followed by filtration through a 0.22 µm nylon membrane to remove particulates. The resulting filtrates were analyzed for flavonoid metabolites using High-Performance Liquid Chromatography-Mass Spectrometry (HPLC-MS).

The HPLC system (Agilent 1200 Series, Agilent Technologies, USA) was coupled to a mass spectrometer (TripleTOF 5600, AB SCIEX, USA). The chromatographic separation was performed on a C18 column (Zorbax Eclipse Plus C18, 4.6 mm × 150 mm, 5 µm particle size, Agilent Technologies). The mobile phase consisted of solvent A (0.1% formic acid in water) and solvent B (0.1% formic acid in acetonitrile). The gradient elution program was as follows: 0-5 min, 5-20% B; 5-10 min, 20-50% B; 10-15 min, 50-90% B; 15-20 min, 90-100% B. The flow rate was set to 0.5 mL/min. Mass spectrometry was performed using electrospray ionization (ESI) in negative ion mode, with the ionization voltage set at -4.5 kV and the source temperature at 500°C. Data were collected in both Full Scan and MS/MS modes, and analyzed using Analyst 1.6.3 and MultiQuant 3.0.3 software. Differential metabolites were identified based on fold change ≥ 2 or fold change ≤ 0.5. Statistical significance was determined using one-way ANOVA (SPSS 26.0, IBM, USA).

#### 2.2.2 Total RNA extraction and high-throughput sequencing

Total RNA was extracted from the exocarp samples using the Plant Total RNA Extraction Kit (Roche, Switzerland), following the manufacturer’s protocol. RNA integrity was assessed by electrophoresis on a 1% agarose gel, while RNA concentration and purity were measured using a NanoPhotometer spectrophotometer (Implen, Germany) and Qubit 2.0 Fluorometer (Thermo Fisher Scientific, USA). The quality of RNA was further confirmed with the Agilent 2100 Bioanalyzer (Agilent Technologies, USA).

RNA samples that passed quality control were used to construct sequencing libraries. Library preparation and sequencing were performed using the Illumina TruSeq RNA Library Prep Kit (Illumina, USA), followed by sequencing on the Illumina NovaSeq 6000 platform (Illumina, USA), generating paired-end 150 bp reads.

#### 2.2.3 Transcriptome data analysis and differentially expressed genes

Raw transcriptomic data were processed for quality control using fastp v0.19.3 (Chen et al., 2018). Clean reads were aligned to the oil palm reference genome (provided by the Oil Palm Genome Database) using HISAT v2.1.0. Gene expression levels were quantified using Fragments Per Kilobase of Transcript Per Million Fragments Mapped (FPKM) calculated by StringTie v1.3.4d. Differentially expressed genes (DEGs) were identified using DESeq2 v1.22.1 with a False Discovery Rate (FDR) < 0.05 and |log2Fold Change| ≥ 1. KEGG pathway enrichment analysis was conducted to identify biological pathways significantly associated with the flavonoid biosynthesis pathway.

#### 2.2.4 Integration of metabolomic and transcriptomic data

Pathways enriched in differentially expressed metabolites and genes were compared against the KEGG database (https://www.genome.jp/kegg). A hypergeometric test was used to identify significant pathways containing more than 25 shared entries, with the top 25 pathways selected based on the lowest p-values. Correlations between genes and metabolites were investigated using Pearson’s correlation analysis (R v4.0.5), with a threshold of correlation coefficient > 0.80 and p-value < 0.05 for selecting significant gene-metabolite relationships.

#### 2.2.5 Statistical analysis

Statistical analyses were conducted using SPSS 26.0 (IBM Corp., USA) and R software (v4.0.5). Data are presented as means ± standard deviation (SD), with statistical significance set at p < 0.05. Differential metabolites were identified based on fold change (FC) ≥ 2 or ≤ 0.5. Differentially expressed genes (DEGs) were identified using DESeq2 with an FDR-adjusted p-value < 0.05 and |log2Fold Change| ≥ 1. Pathway enrichment analysis was performed using the KEGG database.

## 3 Results and analysis

### 3.1 Metabolomic analysis of oil palm exocarp flavonoids

#### 3.1.1 Composition and classification of flavonoids in the oil palm exocarp at different developmental stages

**Figure 2:**
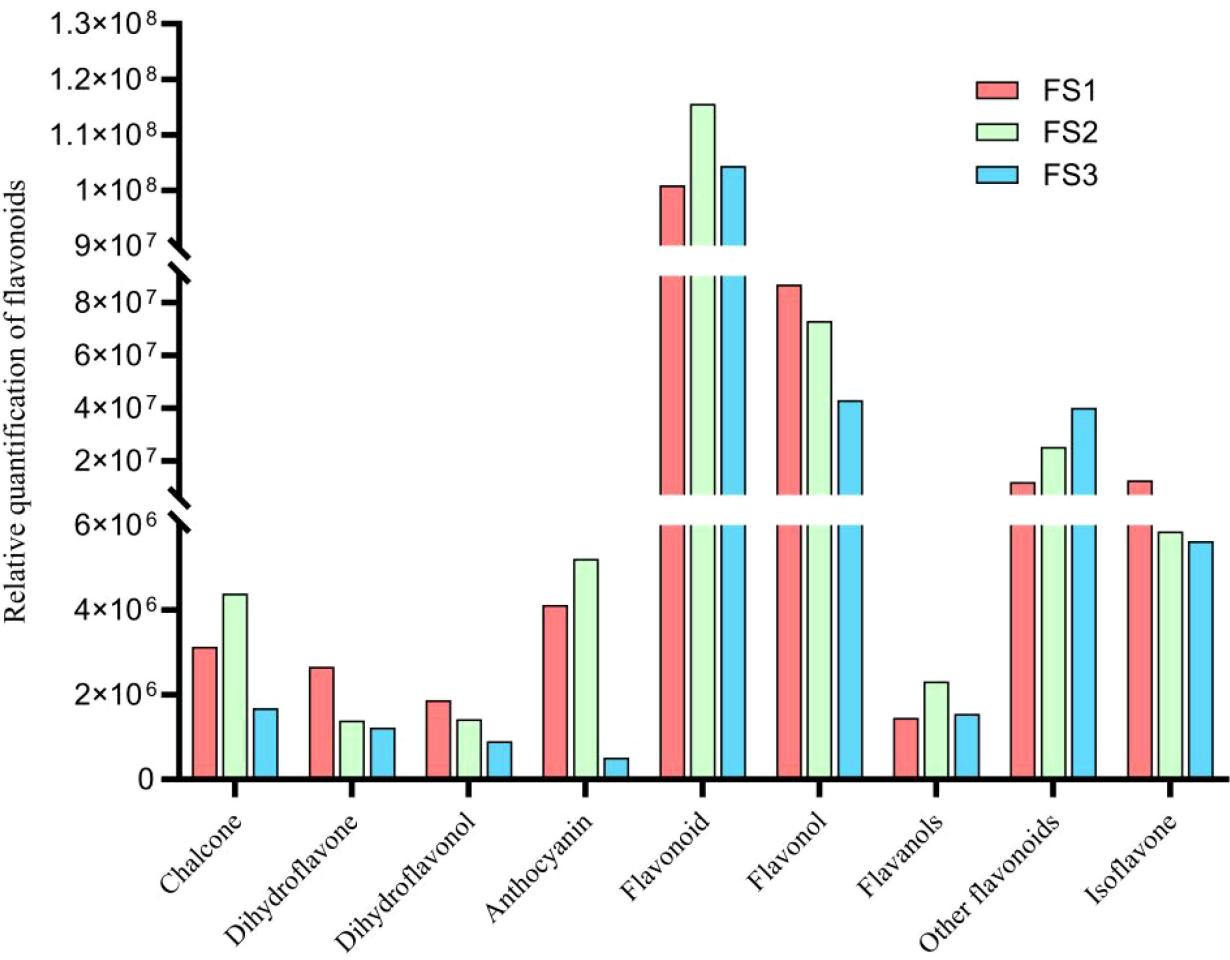
Changes in relative quantification of various flavonoid compounds in the exocarp of oil palm fruits at different developmental periods (FS1-FS3).

**Figure 3:**
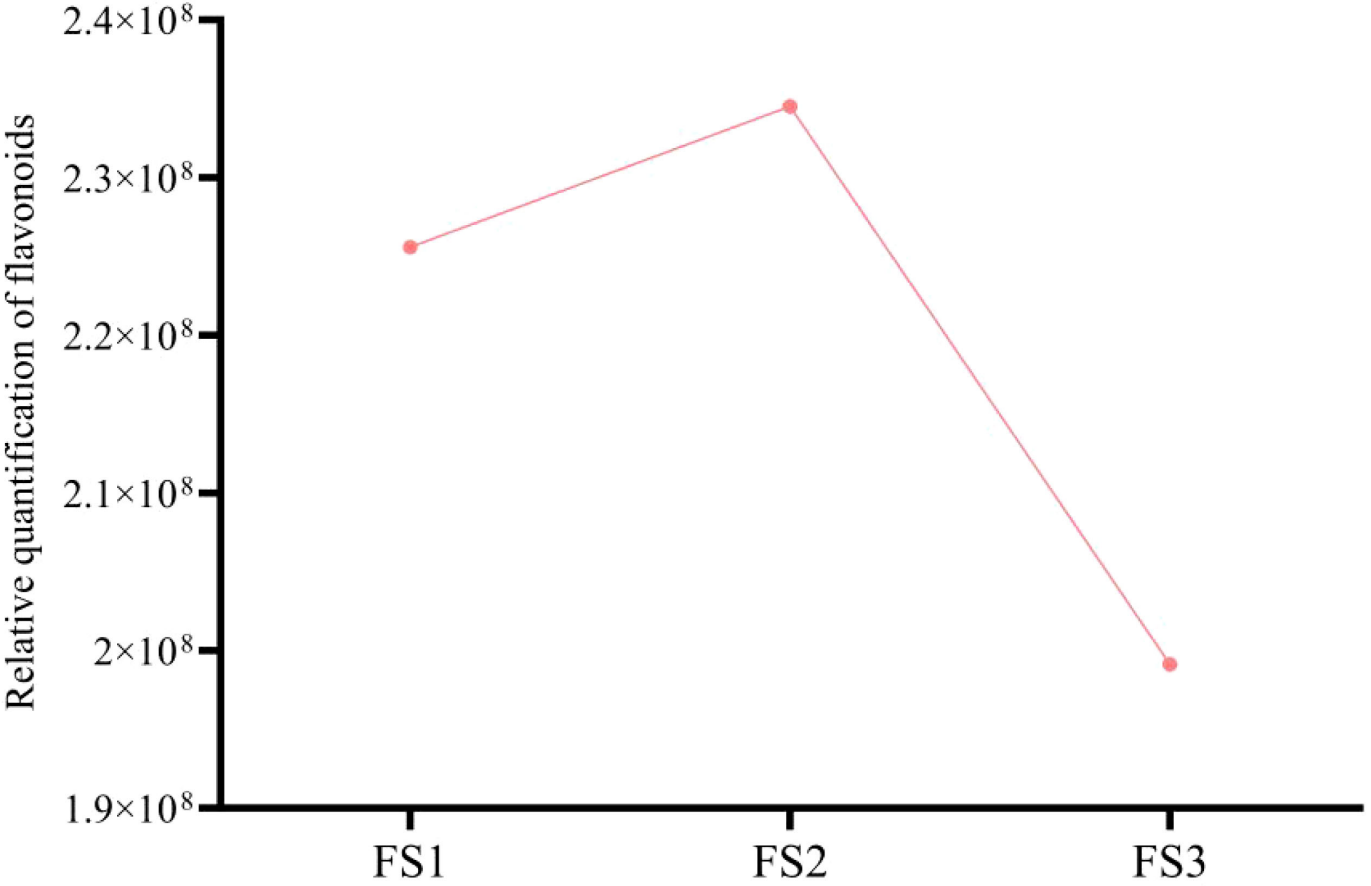
Changes in the total relative quantification of exocarp flavonoid compounds during different developmental periods (FS1-FS3) of oil palm fruits.

**Figure 4:**
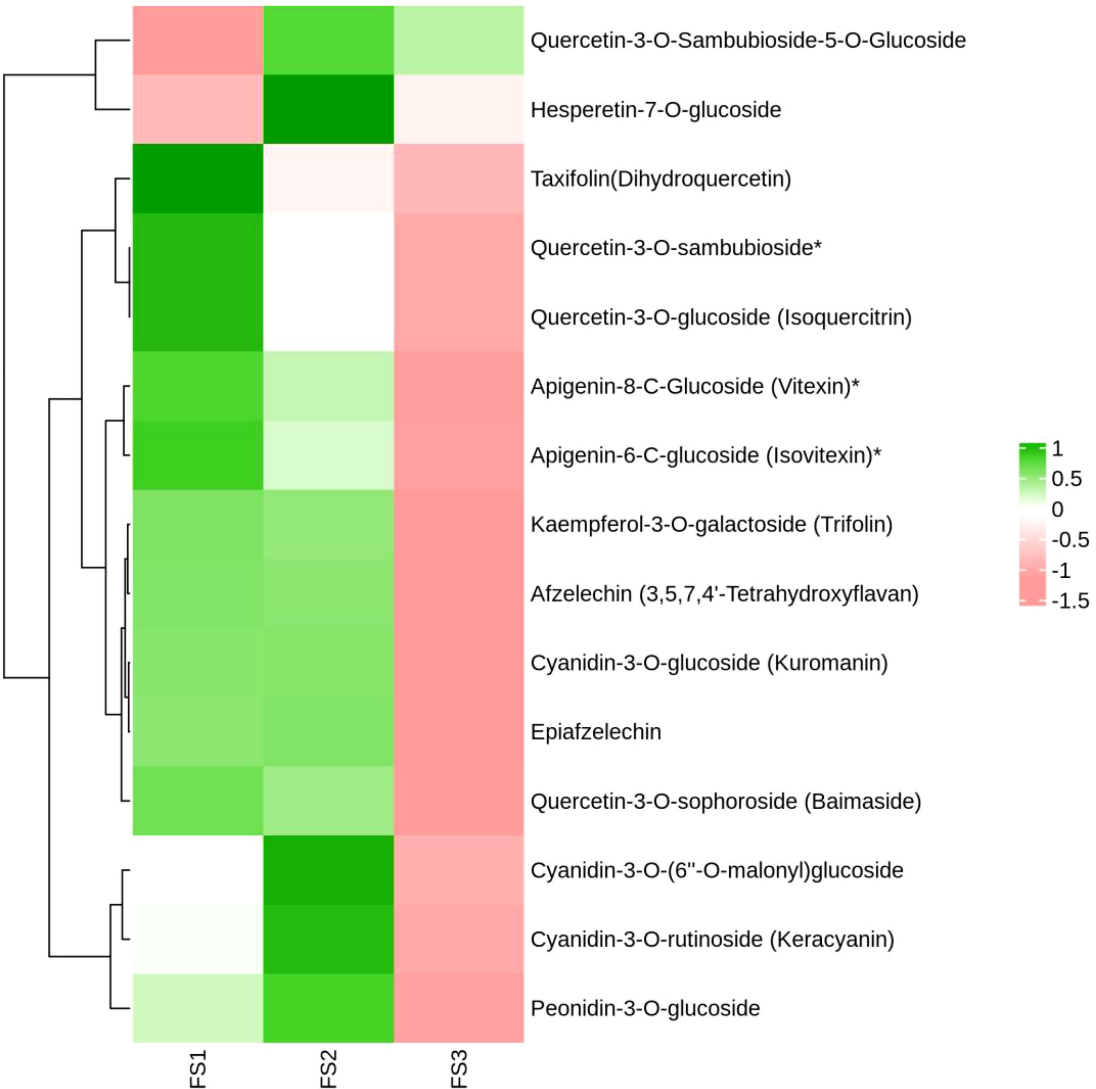
Clustering heat map of relative quantification of exocarp flavonoids in oil palm fruits at different developmental periods (FS1~FS3). (The horizontal coordinates are the different periods of the samples, the vertical coordinates are the differential metabolites, the different colors are the colors filled with the different values obtained from the standardized treatment of different relative contents (green represents high content, red represents low content), the dendrogram on the left side of the heat map represents the results of the hierarchical clustering of the differential metabolites, and the annotated strips on the right side of the clustering map correspond to the substances.)

During the development of the oil palm exocarp, a total of 274 flavonoids were identified and classified into nine distinct groups: 104 flavonoids, 88 flavonols, 7 chalcones, 20 dihydroflavonoids, 6 dihydroflavonols, 9 anthocyanins, 17 flavanols, 15 other flavonoids, and 8 isoflavones. Within these categories, flavonoids exhibited the highest relative content, followed by flavonols, with dihydroflavonols had the lowest relative content. Throughout the developmental stages (FS1 to FS3), the relative contents of flavonoids, chalcones, anthocyanins, and flavanols increased initially before decreasing. In contrast, the relative contents of dihydroflavonoids, dihydroflavonols, flavonols, and isoflavonoids showed a consistent decline, whereas other flavonoids displayed a consistent upward trend (**Figure 2**). The overall relative flavonoid content increased initially and subsequently declined (**Figure 3**). Metabolomic analysis of the FS1 to FS3 developmental stages identified 92 flavonoid metabolites based on screening criteria (Variable Importance in Projection, VIP ≥ 1, Fold_Change ≥ 2, or Fold_Change ≤ 0.5). Of these, 71 metabolites were down-regulated, and 21 were up-regulated. Flavonoids and flavonols comprised the largest proportion of the differential metabolites. Prominent flavonoids included Diosmetin (5,7,3’-Trihydroxy-4’-methoxyflavone), Gnetifolin B, Demethoxysudachitin, Jaceosidin, Vitexin, Isovitexin, and Diosmetin-7-O-rutinoside (Diosmin). Significant flavonols comprised Rhamnocitrin, Rehderianin I, Isorhamnetin-3-O-gallate, and Limocitrin-3-O-galactoside (**Table 1**). The relative content of different flavonoid species in the oil palm exocarp during the FS1-FS3 period was clustered (**Figure 4**). Analysis of the results revealed that the relative content of three flavonoid compounds—Peonidin-3-O-glucoside, Cyanidin-3-O-rutinoside (Keracyanin), and Cyanidin-3-O-glucoside (Kuromanin)—increased from FS1 to FS2, then decreased to FS3, with levels lower than those in FS1.

**Table 1:** Relative quantification of flavonoids in oil palm exocarp across developmental stages.

Compounds	Class	Relative quantification of flavonoids at different developmental periods		
		FS1	FS2	FS3
Apigenin-6-C-glucoside (Isovitexin)*	Flavones	2019620.46	1462332.49	397627.64
5,7,3’,5’-tetrahydroxy-6-methylfavanone	Flavones	93071.33	1824800.72	3941462.31
Diosmetin (5,7,3’-Trihydroxy-4’-methoxyflavone)*	Flavones	91445.73	1890541.58	3870042.83
Apigenin-8-C-Glucoside (Vitexin)*	Flavones	1932329.11	1525720.73	392020.71
5-Hydroxy-6,7,3’,4’-tetramethoxyflavone	Flavones	3621.42	4943.89	17790.89
7,8-Dihydroxy-5,6,4’-trimethoxyflavone	Flavones	6050.10	9531.87	53277.67
6,7,8-Tetrahydroxy-5-methoxyflavone*	Flavones	93548.48	1857192.87	3645527.51
Luteolin-7,3’-di-O-glucoside	Flavones	162889.32	139912.74	8753.91
Chrysoeriol-5-O-glucoside	Flavones	526864.69	293592.78	117382.02
Demethoxysudachitin	Flavones	11691.19	137549.42	436256.74
Luteolin-7-O-(6’’-sinapoyl)glucoside	Flavones	1072242.03	1074463.99	111278.89
4’,5,7-Trihydroxy-3’,6-dimethoxyflavone (Jaceosidin)*	Flavones	13910.22	148175.09	458275.68
5,7-Dihydroxy-3’,4’,5’-trimethoxyflavone	Flavones	9256.37	14342.01	82467.76
Tricin-7-O-(2’’-feruloyl)glucoside	Flavones	705475.59	379078.63	30544.53
Luteolin-8-C-glucoside (Orientin)	Flavones	3564656.88	2605266.90	415992.50
Dihydroxy-dimethoxyflavone*	Flavones	3525.66	6988.13	0
3,5,7,2’-Tetrahydroxyflavone; Datiscetin	Flavones	20328.63	74437.94	122016.34
Gnetifolin B*	Flavones	941174.91	16393090.11	35568924.91
Luteolin-7-O-(6’’-malonyl)glucoside*	Flavones	216449.81	97127.35	23363.70
4’,5-Dihydroxy-3’,5’-dimethoxyflavone	Flavones	8942.47	9446.69	77285.43
Luteolin-6-C-glucoside (Isoorientin)	Flavones	11555755.99	9329137.41	1015143.69
Pedaliin*	Flavones	382916.18	284639.15	86978.89
5,4’-Dihydroxy-3,6,7,3’-tetramethoxyflavone-4’-O-glucoside	Flavones	36036.67	27360.98	3692.06
[6-[2-(3,4-Dihydroxyphenyl)-5,7-dihydroxy-4-oxochromen-3-yl]oxy-3,4,5-trihydroxyoxan-2-yl]methyl acetate*	Flavones	3720338.06	3613463.21	887874.70
Hispidulin-7-O-(6’’-O-p-Coumaroyl)Glucoside	Flavones	97833.66	72511.46	21128.34
3’-O-Methyltricetin-5-O-glucoside*	Flavones	487740.17	469753.54	115474.60
Hispidulin-8-C-glucoside	Flavones	149344.35	119091.50	24789.94
Isosaponarin(Isovitexin-4’-O-glucoside)	Flavones	161405.28	116599.92	32567.78
Tricin-4’-O-glucoside*	Flavones	295466.28	131533.29	66169.24
Luteolin-7-O-gentiobioside	Flavones	149272.48	170131.18	2477.02
Pectolinarigenin*	Flavones	3730.26	6213.40	0
Tricin-7-O-Glucuronide	Flavones	7875.15	7337.83	1392.99
Diosmetin-7-O-rutinoside (Diosmin)*	Flavones	269546.51	254449.49	52761.57
Chrysoeriol-8-C-glucoside (Scoparin)	Flavones	182647.11	110342.50	18930.53
Persicogenin (5,3’-dihydroxy-7,4’-dimethoxyflavanone)	Flavanones	1992.98	0	41497.84
Cirsilineol (4’,5-Dihydroxy-3’,6,7-trimethoxyflavone)	Flavanones	22014.28	41071.45	244873.34
6-C-Glucosyl-2-Hydroxynaringenin	Flavanones	1151798.93	465535.10	224516.94
Aromadendrin-7-O-glucoside*	Flavanonols	885655.49	445495.90	218380.16
Taxifolin(Dihydroquercetin)	Flavanonols	28975.09	14020.04	6989.04
Hesperetin-7-O-glucoside	Flavanones	0	14521.49	4347.09
Taxifolin-3’-O-glucoside	Flavanonols	23067.85	12120.56	5368.11
Cyanidin-3-O-glucosylrutinoside	Anthocyanidins	19352.46	38290.65	6583.67
Peonidin-3-O-rutinoside	Anthocyanidins	154751.58	212483.70	12214.78
Cyanidin-3-O-(6’’-O-p-coumaroyl)glucoside-5-O-glucoside	Anthocyanidins	45218.46	92330.09	11701.11
Cyanidin-3-O-rutinoside (Keracyanin)	Anthocyanidins	1058218.61	1862488.86	148579.56
Cyanidin-3-O-glucoside (Kuromanin)	Anthocyanidins	2343141.57	2362212.82	203875.44
Peonidin-3-O-glucoside	Anthocyanidins	67177.65	94717.09	5368.97
Cyanidin-3-O-(6’’-O-malonyl)glucoside	Anthocyanidins	129740.70	270682.02	21457.54
3,5,4’-Trihydroxy-7-methoxyflavone (Rhamnocitrin)	Flavonols	91664.13	1956973.69	3879757.90
Quercetin-3-O-arabinoside	Flavonols	498076.14	294867.03	85321.06
5,4’-Dihydroxy-3,7-dimethoxyflavone(Kumatakenin)	Flavonols	134861.11	102067.47	966492.75
Morin-3-O-lyxoside	Flavonols	442938.92	285097.41	88199.58
Isorhamnetin; 3’-Methoxy-3,4’,5,7-Tetrahydroxyflavone	Flavonols	86015.33	81902.51	11329.95
Kaempferol-3-O-(6’’-malonyl)glucoside*	Flavonols	173294.87	74263.53	21421.32
Avicularin(Quercetin-3-O-α-L-arabinofuranoside)*	Flavonols	21592.29	10916.24	0
Morin	Flavonols	516432.66	504732.50	104138.66
Quercetin-7-O-(6’’-malonyl)glucoside	Flavonols	732565.40	814849.33	176289.48
Rehderianin I	Flavonols	10467.99	123745.49	368656.02
Kaempferol-7-O-glucoside	Flavonols	375446.56	104576.34	43461.22
Isorhamnetin-3-O-(6’’-acetylglucoside)	Flavonols	613569.29	420904.76	130610.11
Kaempferol-3-O-(6’’-malonyl)galactoside*	Flavonols	166364.22	78066.59	16638.27
Kaempferol-3-O-galactoside (Trifolin)	Flavonols	93021.63	89114.60	17092.06
Isorhamnetin-3-O-(6’’-malonyl)glucoside*	Flavonols	320042.32	262264.19	67188.85
Quercetin-3-O-sambubioside*	Flavonols	25208.77	14377.96	3114.81
Quercetin-3-O-(2’’-O-acetyl)glucuronide	Flavonols	97622.57	69844.51	21525.05
Sexangularetin-3-O-glucoside-7-O-rhamnoside	Flavonols	5471675.85	3281744.78	1200804.43
Kaempferol-3-O-(6’’-O-acetyl)glucoside	Flavonols	689470.35	296202.82	81782.25
8-Methoxykaempferol-7-O-rhamnoside	Flavonols	154721.96	136070.06	20165.95
Quercetin-3-O-glucoside (Isoquercitrin)	Flavonols	101017.68	60105.78	17693.22
Quercetin-3-O-galactoside (Hyperin)*	Flavonols	660741.22	385887.87	109223.27
Isorhamnetin-3-O-gallate	Flavonols	6732.05	20249.67	31132.22
Quercetin-3-O-apiosyl(1→2)galactoside*	Flavonols	23259.11	13121.89	4333.19
Quercetin-3-O-sophoroside (Baimaside)	Flavonols	16808.76	15141.60	1872.83
Isorhamnetin-3-O-arabinoside	Flavonols	4303.43	1477.51	0
Limocitrin-3-O-galactoside	Flavonols	15554.59	17825.99	3745.71
Isorhamnetin-3-O-rutinoside-7-O-rhamnoside*	Flavonols	1112235.95	648935.04	263874.17
Morin-3-O-xyloside*	Flavonols	26754.62	19111.44	4583.89
Catechin-(7,8-bc)-4α-(3,4-dihydroxyphenyl)-dihydro-2-(3H)-one	Flavanols	25419.29	23563.74	6071.30
Epicatechin gallate*	Flavanols	7779.88	82904.80	9679.12
Gallocatechin 3-O-gallate	Flavanols	12158.19	51681.34	34475.50
Afzelechin (3,5,7,4’-Tetrahydroxyflavan)	Flavanols	24295.13	23868.02	4363.36
Epiafzelechin	Flavanols	7921.84	8118.93	0
O-Demethylforbexanthone	Other Flavonoids	499311.66	781147.08	117582.58
1,3,6-trihydroxy-2,5,7-trimethoxyxanthen-9-one	Other Flavonoids	550157.70	141463.00	13894.27
9,11-dimethoxy-2h-[1,3]dioxolo[4,5-b]xanthen-10-one*	Other Flavonoids	973870.96	16543900.64	35670120.09
1,3,6,8-tetrahydroxy-2,5-dimethoxyxanthen-9-one	Other Flavonoids	44424.85	14830.60	4258.32
1,2,4,5-tetrahydroxy-7-(hydroxymethyl)anthracene-9,10-dione	Other Flavonoids	151027.76	75128.35	32235.14
Iristectorigenin A*	Isoflavones	16447.14	139368.46	447987.42
Genistein-8-C-glucoside	Isoflavones	375945.39	279778.97	69260.90
Iristectorin A*	Isoflavones	323003.98	126168.33	69286.04
Genistein-7-O-galactoside*	Isoflavones	5254.35	4093.33	0

The values represent the relative abundance of each compound, measured in arbitrary units, as determined by metabolomics analysis. Compounds marked with an asterisk (*) denote isomeric forms identified during the study. Each compound is categorized into subclasses such as Flavones, Flavanones, Flavanonols, Anthocyanidins, Flavanols, Isoflavones, and Other Flavonoids. This classification highlights the diverse flavonoid profiles across developmental stages, providing insights into their dynamic changes and potential roles during oil palm exocarp development.

(In **Figures 1**–**3** and **Table 1**, the relative quantification is the integrated peak area of the substance in the sample, the peak area is actually the peak area of the peak at the retention time of the quantified ion of the corresponding substance, which represents its specific response intensity, and the higher the intensity of this response, the larger the peak area, which represents the larger the relative content of its substance in the sample.)

The relative contents of the flavonoid compounds Hesperetin-7-O-glucoside and Quercetin-3-O-Sambubioside-5-O-Glucoside were lower in FS1, increased in FS2, and decreased in FS3 although, remained higher than in FS1.Isovitexin*, Vitexin*, Taxifolin (Dihydroquercetin), Kaempferol-3-O-galactoside (Trifolin), *Quercetin-3-O-sambubioside*, and eight other flavonoids showed a continuous decrease in relative content from FS1 to FS3. Cyanidin-3-O-glucoside (Kuromanin) and Epiafzelechin increased in relative content from FS1 to FS2 and decreased in FS3.

#### 3.1.2 Analysis of differential metabolites of flavonoids in the exocarp of oil palm fruits at different developmental periods

**Figure 5:**
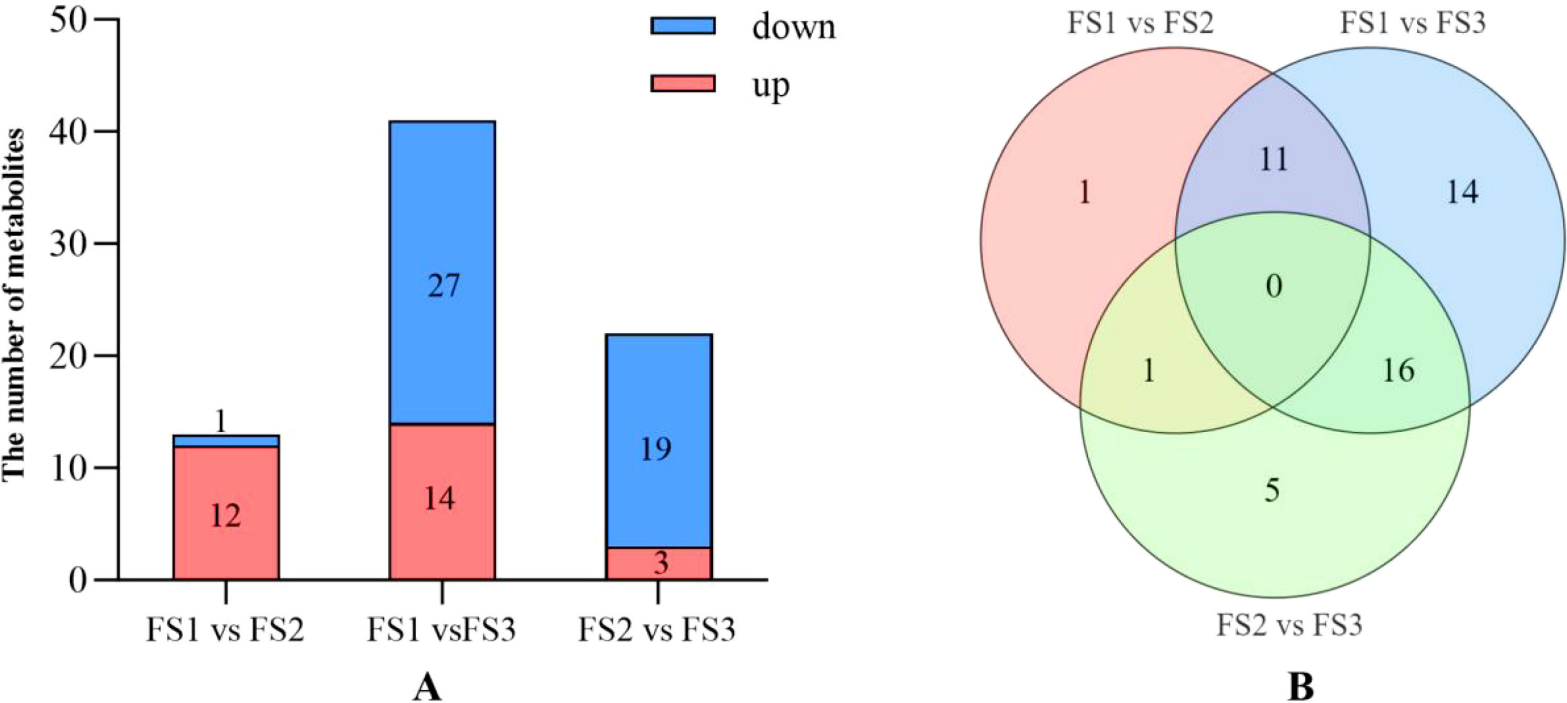
Differential statistics of flavonoid metabolites in the exocarp of oil palm fruits at different developmental stages (FS1-FS3). **(A)** Comparison of the number of upregulated and down-regulated metabolites and the trend of change at each stage of FS1 VS FS2, FS1 VS FS3, FS2 VS FS3. **(B)** Number of the same differential metabolites at each stage of FS1 VS FS2, FS1 VS FS3, FS2 VS FS3.

The number of significantly up-regulated and down-regulated metabolites was determined by analyzing the metabolomic data of oil palm fruits during FS1-FS3 based on the screening criteria (variable importance in projection, VIP) ≥ 1, Fold Change ≥3 or Fold_Change ≤ 0.5 (**Figure 5**). In FS1 vs FS2, there were 12 metabolites showing increased levels, such as hesperetin-7-O-glucoside, diosmetin (5,7,3’-Trihydroxy-4’-methoxyflavone), gnetifolin B, and persicogenin (5,3’-dihydroxy-7,4’-dimethoxyflavanone), along with 1 metabolite showing decreased levels. In the FS1 vs FS3 comparison, hesperetin-7-O-glucoside, 3,5,4’-Trihydroxy-7-methoxyflavone (rhamnocitrin), diosmetin (5,7,3’-trihydroxy-4’-methoxyflavone), and gnetifolin B exhibited decreased levels, while 14 other metabolites showed significant increases. Conversely, isorhamnetin-3-O-arabinoside, epiafzelechin, avicularin (quercetin-3-O-alpha-L-Arabinofuranoside)*, and 27 other metabolites displayed significant decreases. In FS2 vs FS3, three metabolites showed increased levels: persicogenin (5,3’-dihydroxy-7,4’-dimethoxyflavanone), 5,4’-dihydroxy-3,7-dimethoxyflavone (kumatakenin), and 4’,5-dihydroxy-3’,5’-dimethoxyflavone. Meanwhile, the metabolites with decreased levels included epiafzelechin, avicularin (quercetin-3-O-α-L-arabinofuranoside)*, and 19 others. The number of up- and down-regulated differential metabolites initially increased and then decreased as fruit development progressed. No differential metabolites were observed in the comparisons of FS1 vs. FS2, FS1 vs. FS3, and FS2 vs. FS3. A total of 11 common differential metabolites, including Hesperetin-7-O-glucoside, Diosmetin (5,7,3’-Trihydroxy-4’-methoxyflavone), and Gnetifolin B, were identified in both FS1 vs. FS2 and FS1 vs. FS3. Persicogenin (5,3’-dihydroxy-7,4’-dimethoxyflavanone) was a shared differential metabolite in FS1 vs. FS2 and FS2 vs. FS3. Additionally, 16 co-occurring differential metabolites, such as Pectolinarigenin*, Epiafzelechin, and Avicularin (Quercetin-3-O-α-L-arabinofuranoside)*, were found in both FS1 vs. FS3 and FS2 vs. FS3.

### 3.2 Analysis of differential genes in different developmental periods of oil palm pericarp

**Figure 6:**
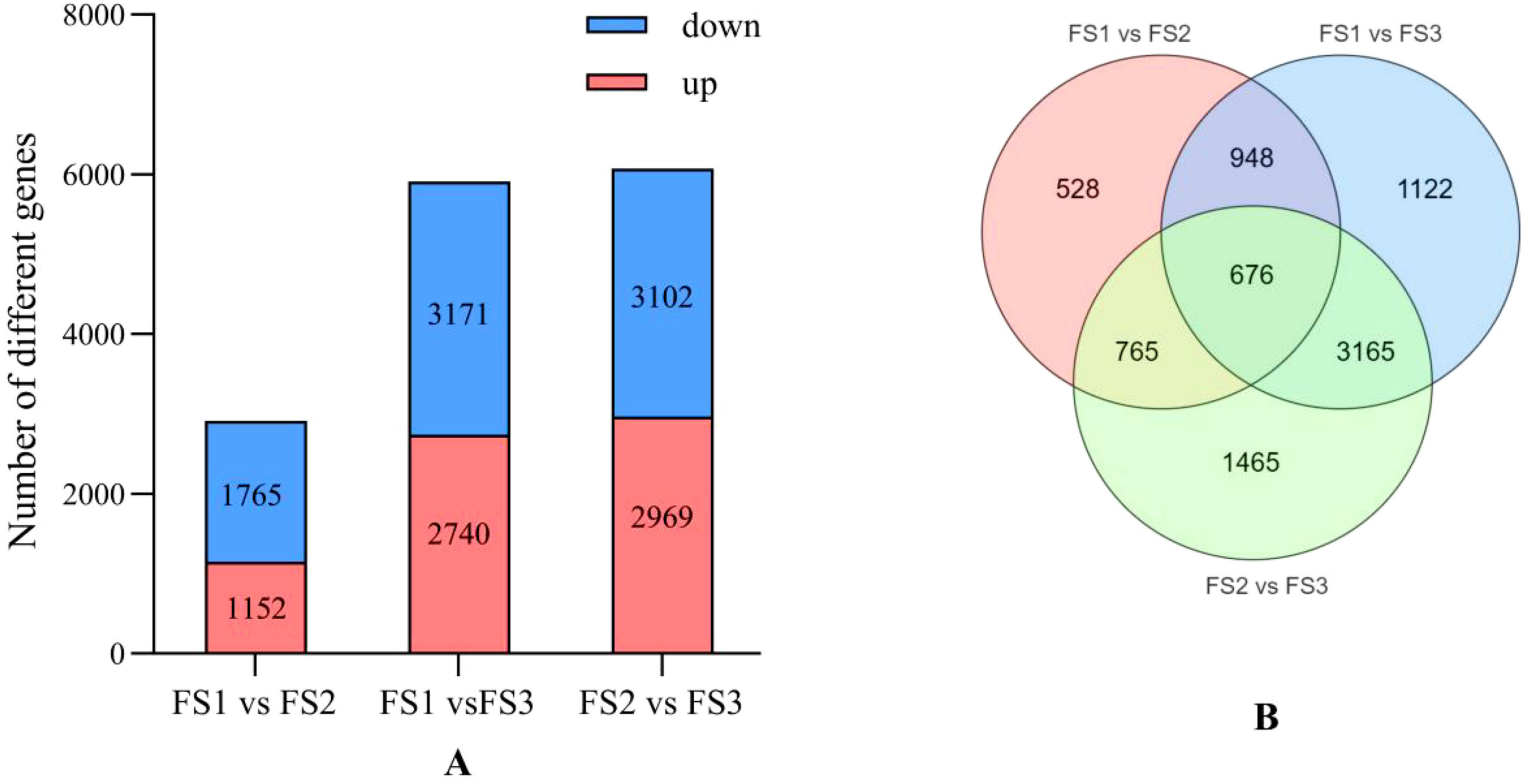
Differential statistics of flavonoid differential genes in the exocarp of oil palm fruits at different developmental stages (FS1-FS3). **(A)** Comparison of up-regulated and down-regulated differential genes and the trend of change at each stage of FS1 VS FS2, FS1 VS FS3, FS2 VS FS3. **(B)** Number of identical differential genes at each stage of FS1 VS FS2, FS1 VS FS3, FS2 VS FS3.

Transcriptome analysis of the oil palm exocarp during developmental stages of FS1, FS2, and FS3 revealed significant differential gene expression. Genes were screened based on criteria of |log2Fold Change| ≥ 1 and FDR < 0.05. In the FS1 vs. FS2 comparison, 1,152 genes were genes (e.g., LOC105048385, LOC105052646, LOC105058257) while1,765 genes were down-regulated (e.g., LOC105049188, LOC105058893, LOC105056795) (**Figure 6A**). For the FS1 vs. FS3 comparison showed 2,740 up-regulated genes (e.g., LOC105032120, LOC105058752, LOC105049762) and 3,171 down-regulated genes (e.g., LOC105038978, LOC105041013, LOC105048687). Whereas, in FS2 vs. FS3 comparison, 2,969 genes were up-regulated (e.g., LOC105041978, LOC105035259, LOC105050452) while 3,102 were down-regulated (e.g., LOC105036076, LOC105035725, LOC105034160). As the fruit development progressed, the number of down-regulated genes consistently exceeded the up-regulated genes, with an overall increase in the counts of both up- and down-regulated genesacross all developmental comparisons (FS1 vs. FS2, FS1 vs. FS3, and FS2 vs. FS3). Across the three developmental stages, 676 differentially expressed genes were identified as common, suggesting shared regulatory pathways or functional roles throughout the fruit’s development (**Figure 6B**).

### 3.3 Joint analysis of metabolic transcriptome data of oil palm fruits at different developmental periods

**Figure 7:**
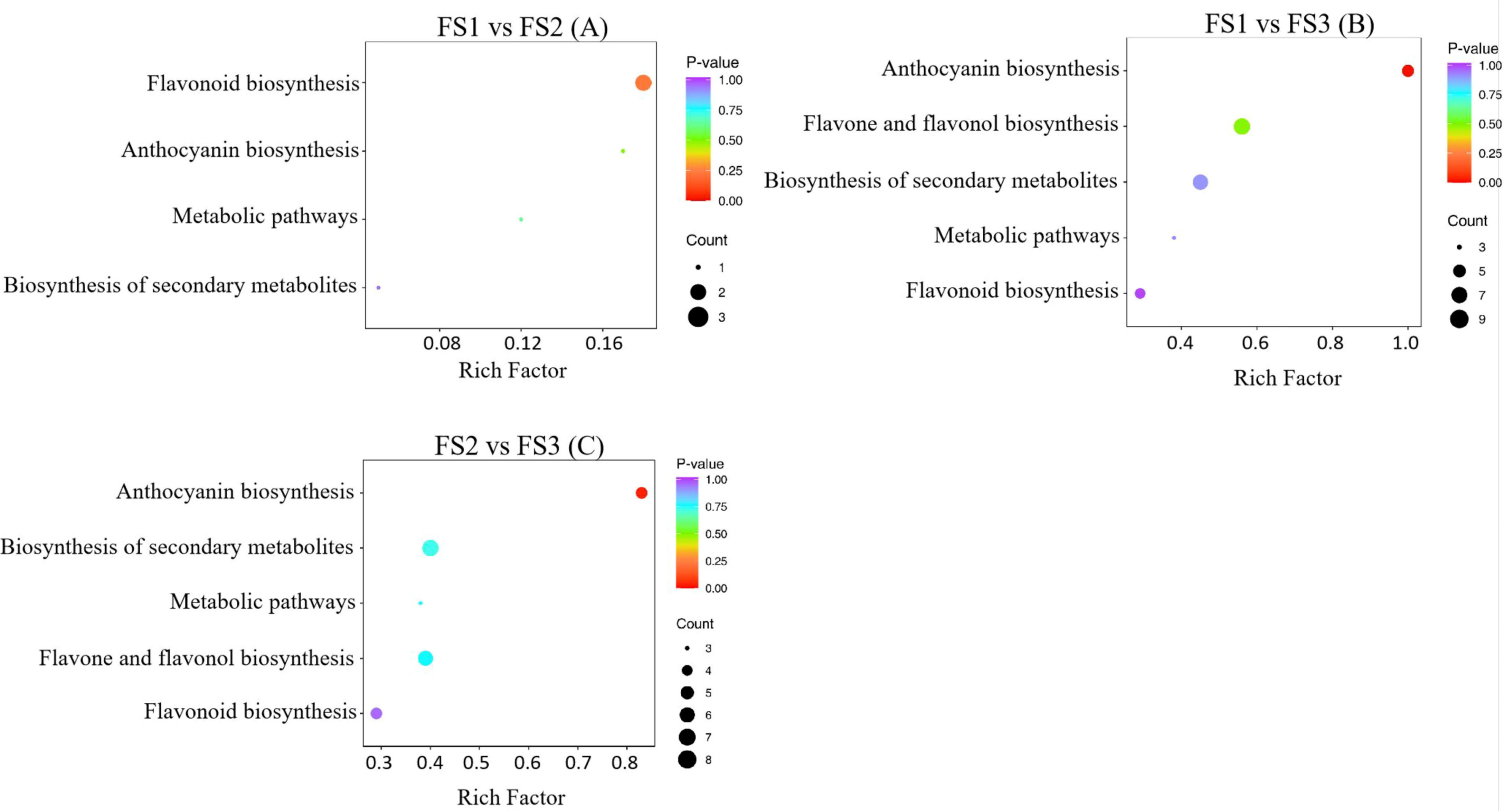
These three images **(A–C)** are described separately in the body of the article, and the images are framed the same way, so they do not need to be described separately again in the tag.

The joint analysis of flavonoid metabolome and transcriptome data from oil palm fruits at various developmental stages revealed that differential metabolites and genes were primarily enriched in five pathways: flavonoid biosynthesis, anthocyanin biosynthesis, flavonoid and flavonol biosynthesis, phytometabolic, and secondary metabolite biosynthesis pathways (**Figure 7**). In the comparison between FS1 and FS2, the flavonoid biosynthesis pathway exhibited the highest number of enriched metabolites (3) and was the most significantly enriched pathway (**Figure 7A**). In FS1 vs. FS3, the anthocyanin biosynthesis pathway showed the most significant enrichment with five metabolites, while the flavonoid and flavonol biosynthesis pathways had the highest number of enriched metabolites but lacked significant enrichment (**Figure 7B**). Similarly, in FS3 vs. FS3, the anthocyanin biosynthesis pathway remained the most significantly enriched, with five metabolites, whereas the flavonoid and flavonol biosynthesis pathway contained six enriched metabolites but was not significantly enriched (**Figure 7C**).

Among the identified pathway, the plant metabolic and secondary metabolite synthesis pathways were identified as the primary routes for synthesis and metabolism in plants, with most metabolites and genes enriched in fruit stages (FS). In the flavonoid biosynthesis pathway (Ko00941) (**Table 2**), metabolites such as Eriodictyol (5,7,3’,4’-Tetrahydroxyflavanone), Afzelechin (3,5,7,4’-Tetrahydroxyflavan), and Epiafzelechin were co-enriched, along with Naringenin-7-O-Neohesperidoside (Naringin)*, Hesperetin-7-O-glucoside, and eight other differential metabolites. Additionally, 47 differentially expressed genes, including LOC105054663, LOC105054281, LOC105035842 were enriched. In the flavonoids and flavonols biosynthesis pathway (Ko00944) (**Table 3**), 10 differential metabolites such as Isovitexin*, Vitexin*, and Vitexin-2’’-O-rhamnoside, as well as 18 differential genes including LOC105036086, LOC105055415, and LOC105058071 were enriched.

**Table 2:** Statistics of differential metabolites and differential genes related to flavonoid biosynthesis pathway (Ko00941) in exocarp of oil palm at different developmental stages (FS1-FS3).

	Differential metabolites	Differential gene ID
FS1 vs FS2	Naringenin-7-O-Neohesperidoside(Naringin)* Taxifolin(Dihydroquercetin) Hesperetin-7-O-glucoside	LOC105054663;LOC105036364;LOC105060373;LOC105054281;LOC105035842;LOC105058232;LOC105058071;LOC105034344
FS1 vs FS3	Apigenin-8-C-Glucoside (Vitexin)*, Taxifolin(Dihydroquercetin), Epiafzelechin, Galangin (3,5,7-Trihydroxyflavone), Hesperetin-7-O-glucoside	LOC105054663;LOC105050451;LOC105036364;LOC105060373;LOC105054281;LOC105035842;LOC105058232;LOC105058071;LOC105052441;LOC105034344 ;LOC105037012
FS2 vs FS3	Apigenin-8-C-Glucoside (Vitexin)*, Taxifolin(Dihydroquercetin), Epiafzelechin, Eriodictyol, Afzelechin	LOC105054663;LOC105052309;LOC105050451;LOC105036364;LOC105060373;LOC105045978;LOC105035842;LOC105058232;LOC105058071;LOC105052441;LOC105037012;LOC105040724;LOC105048473;LOC105041901

* means isomers.

**Table 3:** Statistics of differential metabolites and differential genes related to the biosynthesis pathway of flavonoids and flavonols in exocarpons of oil palm at different developmental stages (FS1-FS3) (Ko00944).

	Differential metabolites	Differential gene ID
FS1 vs FS2	\	\
FS1 vs FS3	Nicotiflorin*,Isovitexin*,3,7-Di-O-methylquercetin,Vitexin*,Vitexin-2’’-O-rhamnoside,Trifolin,Vitexin-2’’-O-rhamnoside,Cynaroside*,Isoquercitrin, Baimaside	LOC105058071;LOC105036086;LOC105041901;LOC105045491;LOC105055415;LOC105057418;LOC105057419
FS2 vs FS3	Isovitexin*,Vitexin*,Vitexin-2’’-O-rhamnoside,Trifolin,Vitexin-2’’-O-rhamnoside,Isoquercitrin, Baimaside	LOC105058071;LOC105036086;LOC105041901;LOC105057418; LOC105036426;LOC105057419

“” indicates that differential metabolites and differential genes on the flavonoid biosynthesis pathway (ko00941) were not detected. * means isomers.

Within the flavonoid biosynthetic pathways, three differential metabolites and eight differential genes were enriched in FS1 VS FS2, five differential metabolites and 11 differential genes in FS1 VS FS3, and five differential metabolites and 14 differential genes in FS2 VS FS3. In the flavonoids and flavonols biosynthesis pathway, no differential metabolites or genes were enriched in FS1 VS FS2, whereas 10 differential metabolites and seven differential genes were enriched in FS1 VS FS3, and seven differential metabolites and six differential genes were enriched in FS2 VS FS3. Based on the results, suggest that the flavonoid biosynthesis pathway, flavonoids and flavonols biosynthesis pathway play important roles in the development and maturation of oil palm fruits, and promote flavonoids synthesis in the exocarp of oil palm during these stages.

**Figure 8:**
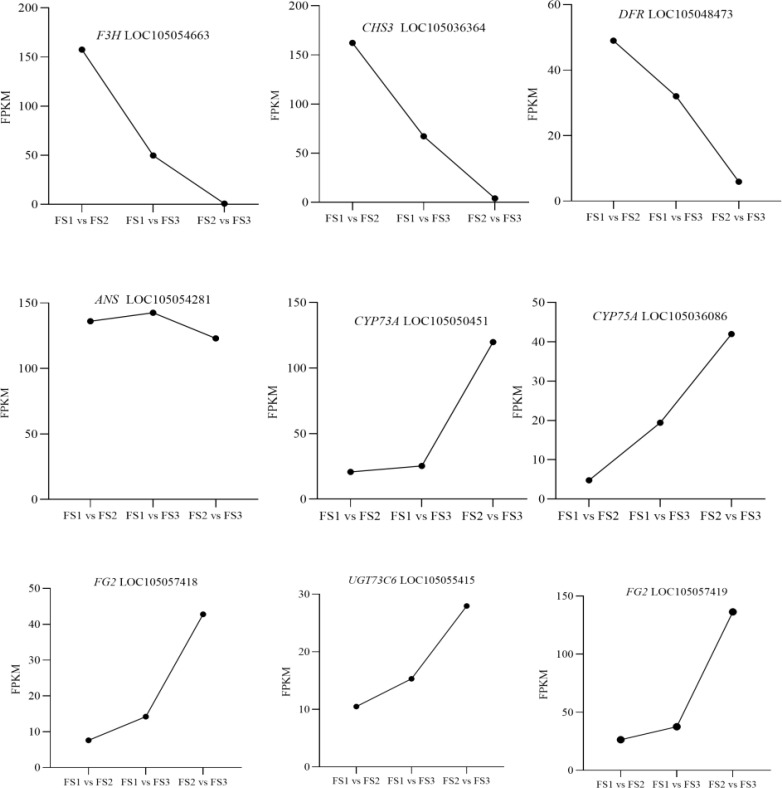
Dynamic changes of the relative contents of key enzyme genes in different stages of oil palm fruit (FS1~FS3). F3H:flavanone 3-hydroxylase, CHS:chalcone synthase, DFR:dihydroflavonol reductase, ANS:anthocyanidin synthase, CYP73A:Cytochrome P450 73A, CYP75A:Cytochrome P450 75A, FG2:flavonol-3-O-glucoside L-rhamnosyltransferase, UTG: UDP-glycosyltransferase,.

In the flavonoid biosynthesis pathway (Ko00941) and the biosynthesis pathway of flavonoids and flavonols (Ko00944), Nr annotation of 65 significantly differentially expressed genes identified nine key enzyme genes—F3H, CHS, ANS, CYP75A, DFR, UGT73C6, and FG2—were highly expressed during oil palm fruit development (**Table 4**). Among the dynamic changes in the expression of these nine key enzyme genes (**Figure 8**), the expression of three genes, LOC105054663, LOC105036364, and LOC105048473 (encoding the enzyme F3H, CHS, and DFR), showed a decreasing trend from FS1-FS3., This trend aligned with the changes in the levels of metabolites such as Afzelechin, Quercetin-3 -O-sambubioside*, and Baimaside. In contrast, the expression of LOC105054281 (encoding ANS) increased initially and then decreased during the FS1-FS3 period, correlating with the fluctuations in the levels of metabolites Epiafzelechin and Hesperetin-7-O-glucoside. The changes in the expression of these nine enzyme genes were correlated with the dynamic fluctuations in the content of the five differential metabolites. Notably the genes LOC105054663, LOC105036364, LOC105054281, and LOC105048473 (enzyme genes: F3H, CHS, ANS, and DFR) showed a significant positive correlation with Quercetin-3-O- sambubioside*. Additionally, LOC105048473 (enzyme gene: DFR) exhibited a significant positive correlation with Afzelechin, Epiafzelechin and Baimaside. Conversley, LOC105054663, LOC105036364, and LOC105054281 (encoding F3H, CHS, ANS) displayed a significant negative correlation with Hesperetin-7-O-glucoside. Furthermore, the genes LOC105050451, LOC105055415, LOC105057418, and LOC105057419 (encoding CYP73A, UGT73C6, FG2-1, FG2-2 respectively) showed a significant negative correlation with Afzelechin, Epiafzelechin, *Quercetin-3-O- sambubioside*, and Baimaside. Finally, LOC105036086 (encoding CYP75A) exhibited a significant negative correlation with Epiafzelechin, Quercetin-3-O-sambubioside*, Baimaside.

**Table 4:** Correlation analysis of key enzyme gene expression levels and relative contents of main flavonoids during the development of oil palm fruit.

Gene Id	Genes	camomile	Epifriedelanol	Hesperidin 7-O-glucoside	Quercetin-3-O-sambubioside*	Baimaside
LOC105054663	*F3H*	0.65	0.73*	-0.88**	0.80**	0.68*
LOC105036364	*CHS*	0.70*	0.79*	-0.83**	0.82**	0.74*
LOC105054281	*ANS*	0.67*	0.76*	-0.87**	0.82**	0.69*
LOC105048473	*DFR*	0.81**	0.88**	-0.69*	0.86**	0.82**
LOC105050451	*CYP73A*	-0.90**	-0.96**	0.41	-0.89**	-0.89**
LOC105036086	*CYP75A*	-0.74*	-0.86**	0.66	-0.90**	-0.80**
LOC105055415	*UGT73C6*	-0.92**	-0.9**	0.58	-0.89**	-0.94**
LOC105057418	*FG2-1*	-0.87**	-0.95**	0.53	-0.90**	-0.85**
LOC105057419	*FG2-2*	-0.89**	-0.97**	0.46	-0.90**	-0.87**

“*” indicates a significant correlation (*P*<0.05), “**” indicates an extremely significant correlation (*P*<0.01).

In summary, F3H, CHS, ANS, and DFR may positively regulate Quercetin-3-O-sambubioside*. DFR may positively regulate the levels of Afzelechin Epiafzelechin and Baimaside. On the other hand, F3H, CHS, and ANS may downregulate the content of Hesperetin-7-O- glucoside. CYP73A, UGT73C6, FG2-1, and FG2-2 may negatively regulate Afzelechin, Epiafzelechin, Quercetin-3-O-sambubioside*, and Baimaside. Finally, CYP75A may downregulate the levels of Epiafzelechin, Quercetin-3-O-sambubioside*, Baimaside.

## 4 Discussions

High-flavonoid oil palm varieties have great nutritional and medicinal potential due to their rich bioactive compounds. Oil palm leaves contain higher polyphenols than green tea, including flavonoids like epigallocatechin and catechin, offering benefits such as protection against cancer, diabetes, hypertension, neurodegenerative diseases, and cardiovascular health (Mohamed, 2014). The Palm Fruit Bioactive Complex (PFBc), derived from palm oil, is rich in antioxidants and polyphenols, which reduce inflammation and improve cognitive function, particularly during aging (Hewlings et al., 2021). Genetic engineering and conventional breeding can enhance the nutritional value of palm oil by increasing carotene and vitamin E content, which provide antioxidative and cardioprotective benefits (Ithnin et al., 2023).

Flavonoids serve diverse functions in plants, including anti-bacterial and antifungal activities, as well as roles in plant coloration, root growth, and pollen development. In this study, we explored the variations in flavonoid accumulation in oil palm at different stages (FS1-FS3) using metabolomics techniques. The results showed that the primary pathways for flavonoid synthesis in oil palm exocarp were the flavonoid biosynthesis pathway and the flavonoid and flavonol biosynthesis pathway. Additionally, the differential metabolite Afzelechin, Epiafzelechin, Hesperetin-7-O-glucoside, Quercetin-3-O-sambubioside*, and Baimaside, were closely associated with flavanoid content in oil palm pericarp.

Transcriptome sequencing is a widely utilized tool in the study of plant flavonoids. CHS is a key enzyme that catalyzes the first critical step in the flavonoid biosynthesis pathway (Wang et al., 2018), while DFR regulates the metabolic flux of flavonoids (Lei et al., 2023). In this study, the expression of CHS and DFR in FS showed a continuous decline, a trend that was consistent with, and positively correlated to the relative content of metabolites such as Afzelechin, Quercetin-3-O-sambubioside*, and Baimaside. These finding suggest that CHS and DFR may play a positive regulatory role in the biosynthesis of flavonoids in oil palm exocarp. Similarly, the expression of F3H in FS also exhibited a continuous decrease, aligning with the relative contents of Afzelechin, Quercetin-3-O-sambubioside*, and Baimaside. A positive correlation was observed between the metabolites Quercetin-3-O-sambubioside* and Baimaside. Among the enzyme genes related to flavonoid synthesis, it was found that CHS and F3H were positively regulated in both the flavonoid biosynthesis and flavonoid and flavonol biosynthesis pathways (Yang et al., 2023). The expression of ANS in FS initially increased and then decreased during fruit ripening, reflecting the changes in the content of Epiafzelechin and Hesperetin-7-O-glucoside. Specifically, ANS expression was positively correlated with Epiafzelechin but negatively correlated with Hesperetin-7-O-glucoside. This pattern aligns with findings in the Azalea cultivar ‘Fenhe’, where down-regulation of ANS was associated with pink coloration (Xia et al., 2022). The expression levels of CYP73A, CYP75A, UGT73C6, and FG-2 in FS were consistently elevated, along with increased contents of Afzelechin, Epiafzelechin, and Quercetin-3-O-sambubioside*, Baimaside. This led to hypothesized that CYP73A, CYP75A, UGT73C6 and FG-2 play a negative regulatory role in the biosynthesis of flavonoids in the oil palm exocarp. In the context of Begonia exocarp browning, CYP73A has been identified as a key gene influencing flavonoid accumulation (Yang et al., 2024a). Meanwhile the enzyme gene CYP75A, and its associated gene family are closely associated to flavonoid biosynthetic enzymes and the regulation of pigments (Yuanyuan et al., 2023). Additionally, UGT73C6, a glycosyltransferase involved in flavonol glycoside biosynthesis in *Arabidopsis thaliana* (Patrik et al., 2003). Genes like FG2 and F3H are highly expressed in sour orange, which biosynthesis fortified flavonoid compounds with enhanced antioxidant activity to detoxify the deleterious effects of reactive oxygen species produced during drought stress (Rao et al., 2023). Flavonoid concentrations, particularly flavonols, are frequently linked to color changes in plants. For instance, in hibiscus, flavonol concentrations exhibit an inverse relationship with anthocyanin levels, playing a significant role in flower coloration and antioxidant activity (Mejía et al., 2023). In oil palm, previous research has distinguished between black-fruited and green-fruited varieties, with black coloration attributed to anthocyanins and carotenoids contributing to fruit coloration in both varieties (Suraninpong and Nuanlaong, 2022). This study revealed that the ratio of flavonol content to total flavonoid content in the oil palm pericarp ranged from 21.62% to 38.45%. Although few studies have explored flavonols in oil palm, the findings suggest that high flavonol content may contribute to color changes in the exocarp during fruit development. Therefore, hypothesized that a high content of flavonols may be related to color changes in FS oil palm exocarp.

The insights gained from this study have potential applications in the development of high-flavonoid oil palm varieties. Enhanced flavonoid content could improve the nutritional value of oil palm products. Further research could focus on manipulating key regulatory genes, such as CHS, F3H, DFR, and ANS, through genetic engineering or marker-assisted selection to achieve these goals. By advancing our understanding of flavonoid biosynthesis in oil palm, this study lays the groundwork for practical applications in agriculture, food, and biotechnology.

## 5 Conclusions

Study provides new insights into the flavonoid metabolism in oil palm, specifically in the mesocarp and exocarp. As the fruit develops and ripens, the relative content of flavonoids initially increases and then decreases, which may be influenced by the flavonoid biosynthetic pathway, enzyme gene regulation, and differential metabolites. Key genes such as F3H, CHS, ANS, and DFR are likely involved in promoting the synthesis of flavonoids like quercetin-3-O-sambubioside, afzelechin, epiafzelechin, and baimaside. Conversely, genes such as CYP73A, CYP75A, UGT73C6, and FG2 appear to suppress the synthesis of these flavonoids, suggesting complex regulatory mechanisms in the biosynthesis of flavonoids in oil palm fruits.

The findings of this study offer valuable genetic insights into the regulation of flavonoid synthesis and metabolism, laying the groundwork for future research into the molecular mechanisms underlying flavonoid biosynthesis in oil palm. To deepen our understanding, further investigations are needed to explore the functional roles of the nine key enzyme genes identified—F3H, CHS, ANS, DFR, CYP73A, CYP75A, UGT73C6, FG2-1, and FG2-2. Future work should include functional experiments to confirm these gene functions and examine their interactions with transcription factors. Ultimately, this research could contribute to the development of oil palm varieties with enhanced flavonoid content, offering new opportunities for product development and the sustainable utilization of oil palm.

## Data Availability

The datasets presented in this study can be found in online repositories. The names of the repository/repositories and accession number(s) can be found in the article/**Supplementary Material**. The data presented in the study are deposited in the NCBI repository, accession number PRJNA1236833.

## References

[B1] Casas M. I. Duarte S. Doseff A. I. Grotewold E. (2014). Flavone-rich maize: an opportunity to improve the nutritional value of an important commodity crop. Front. Plant Sci. 5, 440. doi: 10.3389/fpls.2014.00440 25250036 PMC4157551

[B2] Chen S. Zhou Y. Chen Y. Gu J. (2018). fastp: an ultra-fast all-in-one FASTQ preprocessor Vol. 34 (Oxford, England: Bioinformatics), i884–i890. 10.1093/bioinformatics/bty560 PMC6129281 30423086

[B3] Falcone Ferreyra M. L. Rius S. P. Casati P. (2012). Flavonoids: biosynthesis, biological functions, and biotechnological applications. Front. Plant Sci. 3. doi: 10.3389/fpls.2012.00222 PMC3460232 23060891

[B4] Guo X. Y. Lv Y. Q. Ye Y. Liu Z. Y. Zheng X. Q. Lu J. L.. (2021). Polyphenol oxidase dominates the conversions of flavonol glycosides in tea leaves. Food Chem. 339, 128088. doi: 10.1016/j.foodchem.2020.128088 32979714

[B5] Hewlings S. J. Draayer K. Kalman D. S. (2021). Palm Fruit Bioactive Complex (PFBc), a source of polyphenols, demonstrates potential benefits for inflammaging and related cognitive function. Front. Plant Sci. 13, 1127. doi: 10.3390/nu13041127.13 PMC8066389 33808068

[B6] Ithnin M. Othman A. Tahir N. I. M. Banisetti K. B. Abd Halim M. A. Rajesh M. K. (2023). “Oil palm: Genome designing for improved nutritional quality,” in Compendium of crop genome designing for nutraceuticals. Ed. Kole C. (Springer Nature Singapore, Singapore), 1–41. doi: 10.1007/978-981-19-4169-6_22

[B7] Jiu S. Guan L. Leng X. Zhang K. Haider M. S. Yu X.. (2022). The role of VvMYBA2r and VvMYBA2w alleles of the MYBA2 locus in the regulation of anthocyanin biosynthesis for molecular breeding of grape (Vitis spp.) skin coloration. Plant Biotechnol. J. 19, 1216–1239. doi: 10.1111/pbi.13543 PMC8196647 33440072

[B8] John Martin J. J. Yarra R. Wei L. Cao H. (2022). Oil palm breeding in the modern era: challenges and opportunities. Plants 11, 1395. doi: 10.3390/plants11111395 35684168 PMC9183044

[B9] Jones P. Messner B. Nakajima J. I. Schaffner A. R. Saito K. (2003). UGT73C6 and UGT78D1, glycosyltransferases involved in flavonol glycoside biosynthesis in Arabidopsis thaliana. J. Biol. Chem. 278, 43910–43918. doi: 10.1074/jbc.m303523200 12900416

[B10] Kalt W. Cassidy A. Howard L. R. Krikorian R. Stull A. J. Tremblay F.. (2020). Recent research on the health benefits of blueberries and their anthocyanins. Adv. Nutr. (Bethesda Md.) 11, 224–236. doi: 10.1093/advances/nmz065 PMC7442370 31329250

[B11] Khodadadi F. Tohidfar M. Vahdati K. Dandekar A. M. Leslie C. A. (2020). Functional analysis of walnut polyphenol oxidase gene (JrPPO1) in transgenic tobacco plants and PPO induction in response to walnut bacterial blight. Plant Pathol. 69, 756–764. doi: 10.1111/ppa.13159

[B12] Lai J. Li C. Zhang Y. Wu Z. Li W. Zhang Z.. (2023). Integrated transcriptomic and metabolomic analyses reveal the molecular and metabolic basis of flavonoids in areca catechu L. J. Agric. Food Chem. 71, 4851–4862. doi: 10.1021/acs.jafc.2c08864 36940468

[B13] Lan X. Yang J. Abhinandan K. Nie Y. Li X. Li Y.. (2017). Flavonoids and ROS play opposing roles in mediating pollination in ornamental kale (Brassica oleracea var. acephala). Mol. Plant 10, 1361–1364. doi: 10.1016/j.molp.2017.08.002 28827168

[B14] Lei T. Huang J. Ruan H. Qian W. Fang Z. Gu C.. (2023). Competition between FLS and DFR regulates the distribution of flavonols and proanthocyanidins in Rubus chingii Hu. Front. Plant Sci. 14, 1134993. doi: 10.3389/fpls.2023.1134993 36968391 PMC10031046

[B15] Li Y. Zhao X. Zhang M. M. He X. Huang Y. Ahmad S.. (2023). Genome-based identification of the CYP75 gene family in Orchidaceae and its expression patterns in Cymbidium goeringii. Front. Plant Sci. 13, 1243828. doi: 10.3389/fpls.2023.1243828 PMC10564990 37828920

[B16] Mejía J. J. Sierra L. J. Ceballos J. G. Martínez J. R. Stashenko E. E. (2023). Color, antioxidant capacity and flavonoid composition in hibiscus rosa-sinensis cultivars. Molecules 28, 1779. doi: 10.3390/molecules28041779 36838766 PMC9960340

[B17] Mohamed S. (2014). Oil palm leaf: A new functional food ingredient for health and disease prevention. Front. Plant Sci. 5. doi: 10.4172/2157-7110.1000300

[B18] Murata K. Kitano T. Yoshimoto R. Takata R. Ube N. Ueno K.. (2020). Natural variation in the expression and catalytic activity of a naringenin 7-O-methyltransferase influences antifungal defenses in diverse rice cultivars. Plant J. 101, 1103–1117. doi: 10.1111/tpj.14577 31630460

[B19] Rao M. J. Feng B. Ahmad M. H. Tahir ul Qamar M. Aslam M. Z. Khalid M. F.. (2023). LC-MS/MS-based metabolomics approach identified novel antioxidant flavonoids associated with drought tolerance in citrus species. Front. Plant Sci. 14, 1150854. doi: 10.3389/fpls.2023.1150854 37636085 PMC10450343

[B20] Sadat-Hosseini M. Bakhtiarizadeh M. R. Boroomand N. Tohidfar M. Vahdati K. (2020). Combining independent *de novo* assemblies to optimize leaf transcriptome of Persian walnut. PloS One 15, e0232005. doi: 10.1371/journal.pone.0232005 32343733 PMC7188282

[B21] Shen N. Wang T. Gan Q. Liu S. Wang L. Jin B. (2022). Plant flavonoids: Classification, distribution, biosynthesis, and antioxidant activity. Food Chem. 383, 132531. doi: 10.1016/j.foodchem.2022.132531 35413752

[B22] Suraninpong P. Nuanlaong S. (2022). Comparative transcriptome profiling and molecular marker development for oil palm fruit color. Sci. Rep. 12, 15507. doi: 10.1038/s41598-022-19890-2 36109663 PMC9478095

[B23] Tan H. Man C. Xie Y. Jun Y. J. Fang C. J. (2019). A crucial role of GA-regulated flavonol biosynthesis in root growth of Arabidopsis. Mol. Plant 12, 521–537. doi: 10.1016/j.molp.2018.12.021 30630075

[B24] Testai L. Calderone V. (2017). Nutraceutical value of citrus flavanones and their implications in cardiovascular disease. Nutrients 9, 502. doi: 10.3390/nu9050502 28509871 PMC5452232

[B25] Wang Z. Yu Q. Shen W. El-Mohtar C. A. Chun Z. X. Gmitter F. J. (2018). Functional study of CHS gene family members in citrus revealed a novel CHS gene affecting the production of flavonoids. BMC Plant Biol. 18, 1–13. doi: 10.1186/s12870-018-1418-y 30208944 PMC6134715

[B26] Xia X. Gong R. Zhang C. (2022). Integrative analysis of transcriptome and metabolome reveals flavonoid biosynthesis regulation in Rhododendron pulchrum petals. BMC Plant Biol. 22, 401. doi: 10.1186/s12870-022-03762-y 35974307 PMC9380304

[B27] Yang N. (2023). Joint transcriptome and metabolome analysis of flavonoid content differences during the growth of golden sunflower Anemones (Inner Mongolia Agricultural University). doi: 10.27229/d.cnki.gnmnu.2023.001245

[B28] Yang C. Sun N. Qin X. Liu Y. Sui M. Zhang Y.. (2024a). Multi-omics analysis reveals the biosynthesis of flavonoids during the browning process of Malus sieversii explants. Physiologia Plantarum 176, e14238. doi: 10.1111/ppl.14238 38488414

[B29] Yang C. Zhang S. John Martin J. J. Fu X. Li X. Cheng S.. (2024b). An in-depth study of anthocyanin synthesis in the exocarp of virescens and nigrescens oil palm: metabolomic and transcriptomic analysis. BMC Plant Biol. 24, 910. doi: 10.1186/s12870-024-05607-2 39349997 PMC11441260

[B30] Zain M. S. C. Yeoh J. X. Lee S. Y. Afzan A. Shaari K. (2021). Integration of choline chloride-based natural deep eutectic solvents and macroporous resin for green production of enriched oil palm flavonoids as natural wound healing agents. Antioxidants 10, 1802. doi: 10.3390/antiox10111802 34829674 PMC8615239

[B31] Zhang M. Zhang J. Xiao Q. Li Y. Jiang S. (2024). Reduction of flavonoid content in honeysuckle via Erysiphe lonicerae-mediated inhibition of three essential genes in flavonoid biosynthesis pathways. Front. Plant Sci. 15. doi: 10.3389/fpls.2024.1381368 PMC11059088 38689843

[B32] Zhou L. Sun X. Iqbal A. Yarra R. Wu Q. Li J.. (2024). Revealing the aromatic sonata through terpenoid profiling and gene expression analysis of aromatic and non-aromatic coconut varieties. Int. J. Biol. Macromolecules 280, 135699. doi: 10.1016/j.ijbiomac.2024.135699 39288860

